# Gastrointestinal Diseases in Children Living with HIV

**DOI:** 10.3390/microorganisms9081572

**Published:** 2021-07-23

**Authors:** Francesca Wanda Basile, Maria Cristina Fedele, Andrea Lo Vecchio

**Affiliations:** 1 Baylor International Pediatric AIDS Initiative, Pediatrics, Baylor College of Medicine, 1100 Bates Street, Houston, TX 77030, USA; francescawbasile@gmail.com; 2 Department of Woman, Child and of General and Specialized Surgery, University of Campania “Luigi Vanvitelli”, Via Luigi De Crecchio 2, 80138 Naples, Italy; cristina.fedele90@gmail.com; 3 Department of Translational Medical Science, Section of Pediatrics—University of Naples Federico II, 80131 Naples, Italy

**Keywords:** HIV, pediatric, diarrhea, vomiting, opportunistic, microbiota, ART, pancreatitis, *H. pylori*, endoscopy

## Abstract

Thanks to the advances in antiretroviral therapies (ART) and early diagnosis, pediatric HIV has turned into a chronic infection that requires the collaboration of all pediatric subspecialists for holistic patient management. Gastrointestinal complaints are a frequent reason for seeking access to medical care in all pediatric patients worldwide. Intestinal involvement is present in virtually all children with HIV infections. In high-prevalence settings, up to 25% of children accessing the hospital for diarrhea are diagnosed with HIV. More than half of patients with advanced disease present with gastrointestinal symptoms, from mild infectious diarrhea to severe gastrointestinal impairment, malabsorption and failure to thrive. Gastrointestinal disorders do not spare children on ART, particularly in the initial months of therapy. ART-associated pancreatitis and hepatitis are rare but potentially severe adverse events, whereas lower abdominal symptoms have been reported in more than a third of patients. The latter are usually mild and transient, but may limit ART adherence; a correct framing of the problem is necessary to minimize therapy switches while optimizing the quality of life of children on ART. This review aims to provide state-of-the-art guidance for the initial approach to gastrointestinal diseases in children living with HIV.

## 1. HIV Infection, the Intestinal Barrier, and ART: A Complex Interplay

Human immunodeficiency virus (HIV)-induced intestinal damage is transversal and affects virtually all components of the intestinal epithelium via direct cytopathic and indirect (immune-mediated) mechanisms. HIV attacks gut-associated lymphoid tissue (GALT) in the early stages of the acute infection, targeting preferentially activated memory T cells expressing C-C chemokine receptor 5 (CCR5), which are largely represented in the intestinal mucosa [1].

In parallel, in response to the infection, the increase in pro-inflammatory cytokines such as IL-6, IL-10, and IFN-γ in the lamina propria contributes to the depletion of mucosal Th17, a CD4+ T-cell population that is heavily involved in maintaining gut homeostasis [2,3].

In the 1990s, at the peak of the acquired immune deficiency syndrome (AIDS) epidemic, several ex vivo studies found HIV-RNA in different intestinal cell subsets at the base of the crypts and within the lamina propria at all stages of the disease, and demonstrated villous atrophy, crypt distortion, a decrease in the crypt/villus ratio, and abnormal lymphocyte infiltrates within the lamina propria [4,5,6]. In addition to this, the virus disrupts the integrity of the intestinal wall, promoting enterocyte apoptosis and attacking tight and adherens junctions [7].

The subversion of the intestinal architecture and HIV-associated intestinal immune damage, involving GALT (and particularly Th17 cells) impoverishment and cytokine imbalances, are thought to have a key role in microbial translocation in HIV. The phenomenon of microbial translocation occurs when microorganisms or immunogen bacterial components, such as lipopolysaccharide (LPS) and 16S ribosomal DNA (rDNA), find a way from the intestinal lumen into systemic circulation through the leaky epithelial barrier. In HIV-infected subjects, microbial translocation has been associated with worse health outcomes and cellular/soluble immune activation pathways. In patients on ART, higher plasmatic levels of LPS/16S rDNA are associated with raised systemic inflammatory biomarkers and poorer immune function restoration [8].

Microbial translocation is therefore seen as a contributing factor to the chronic inflammatory status that is the hallmark of people living with HIV despite a good virologic response to ART [9,10,11]. Many clinical studies have shown that antiretrovirals, even when started immediately, can only partially overcome the early structural damage or restore intestinal immune functions, and a subclinical impairment of GALT has been documented in up to one third of HIV-infected patients on treatment, regardless of the ART regimen [12]. However, most findings come from old cohorts of ART-experienced teenagers and adults with complex drug histories, and only recent research has focused on “tested and treated” groups, hypothesizing that a well-timed commencement of antiretrovirals may contribute to decreasing the proviral reservoir in GALT and reducing the long-term consequences of the intestinal damage [13].

In fact, in contrast with adults, vertically infected children started on ART soon after birth exhibit not only a smaller proviral reservoir in GALT, but also progressively lower levels of chronic inflammation and microbial translocation biomarkers, suggesting that timely ART in the acute phases of the infection is effective in limiting the intestinal damage. In particular, recent studies have questioned the model that links microbial translocation to immune activation, although pediatric data are still somewhat discordant or inconclusive, and longitudinal studies with longer follow-ups are needed to further clarify these aspects [14,15,16].

At the other end of the spectrum, the destruction of intestinal barrier integrity, which is associated with uncontrolled viral replication and advanced disease, constitutes so-called “HIV enteropathy”. When ART is not promptly initiated, in most cases there will be a progression to extensive epithelial damage and villous atrophy. Aside from the classical picture of enterocyte disruption, other common histological findings include inflammatory infiltrates of lymphocytes, crypt hyperplasia, and villous blunting [4,5]. The clinical features of HIV enteropathy are those of a malabsorption disorder due to chronic intestinal failure: increased intestinal permeability, bile acid, and micronutrient malabsorption, lactose intolerance, protein-losing enteropathy, and eventually failure to thrive [17,18]. Severe diarrhea is not a constant, although steatorrhea is virtually always present. Idiopathic HIV enteropathy is defined by the absence of any GI pathogen despite extensive microbiological investigations, but in advanced HIV disease, it is common to see an overlapping of this condition with opportunistic intestinal infections. The mainstays of the management of HIV enteropathy are nutritional support and the prompt initiation of effective ART [19].

## 2. Intestinal Microbiota Changes in HIV Infection: A Chance for New Therapeutic Approaches?

The imbalances caused by HIV infection can alter the composition of the intestinal microbiota, favoring a permissive environment for the proliferation of pathogenic bacteria (pathobionts) [20].

Drugs can affect the gut microbial composition, but evidence on the direct effect of ART on the microbiome is limited, and results are affected by several confounding factors. It seems that protease inhibitor (PI)-based regimens have the most harmful effect [21]. Cotrimoxazole, recommended by several clinical practice guidelines for the prophylaxis of *P. jiroveci* infection, does not seem to alter the microbiota, while exerting an antibiotic effect on gut-resident streptococci, reducing intestinal and systemic inflammation [22].

In HIV-infected children, it is common to observe a reduction in Firmicutes (lactobacilli, streptococci, staphylococci, and clostridia) and an increase in *Prevotella* and Enterobacteriaceae, which have often been associated with microbial translocation, especially in children with low CD4 counts. These changes appear to be constant in cohorts of patients from different settings. As seen with other expressions of HIV-induced intestinal damage, long-course ART may counteract dysbiosis, although pediatric evidence is still somewhat limited and discordant [23,24,25,26].

In vitro evidence has demonstrated a link between dysbiosis, microbial translocation, and the systemic pro-inflammatory status seen in vertically infected children. New ways of modulating the gut microbiota in HIV are being explored as potential therapeutical approaches to reduce intestinal inflammation, and also at the subclinical level [27,28,29].

A short trial with a prebiotic/probiotic combination in a cohort of perinatally-infected children was effective to reduce dysbiosis, and a prolonged administration was associated with increased immune reconstitution and anti-inflammatory effects. Similarly, a multicenter randomized controlled trial showed that a prebiotic/probiotic mixture promoted the growth of beneficial gut bacteria in healthy HIV-exposed infants. Still, more data from larger randomized controlled trials on children living with HIV are needed [30,31,32,33,34].

## 3. Upper Gastrointestinal Diseases

Children living with HIV, either on ART or not, often suffer from symptoms associated with upper GI involvement.

### 3.1. ART-Naïve Children and Advanced Disease

Oral thrush, dysphagia, difficulty swallowing, vomiting, and dyspepsia are the most frequent upper GI complaints in older children. In contrast, odynophagia, leading to food refusal and weight loss, can be the only presenting sign in small infants [35,36].

The oral cavity, esophagus, and stomach are frequent targets in HIV/AIDS and associated opportunistic infections (OI) in children with advanced disease. The degree of immunosuppression is the primary determinant for OI, which are reported in almost two-thirds of children with low CD4+ counts [37]. For many years, *Candida* infection was thought to be the primary cause of gastroesophageal disorders in this population, but after the introduction of routine GI endoscopy, cytomegalovirus (CMV), herpes simplex virus (HSV), and idiopathic esophageal ulcers have emerged as leading etiologies of upper GI morbidity. In particular, chronic CMV and *Candida* can cause severe ulceration and esophageal stricture, requiring invasive therapeutic approaches [38]. Idiopathic esophageal ulcers are probably immunologically-mediated manifestations, triggered by HIV infection [39,40,41].

In patients with severe immunosuppression, oral candidiasis may be used as a proxy for esophageal involvement; however, in consideration of the diversity of etiologies, gastric endoscopy—rather than empirical treatment with fluconazole—is currently highly recommended in all such children presenting with oropharyngeal thrush, especially if associated with fever, loss of appetite, dysphagia, or incoercible vomiting. Endoscopy is also helpful for diagnosing other opportunistic causes of chronic esophagitis, such as HSV, CMV, and *Mycobacterium avium–intracellulare* complex (MAC).

In high-prevalence settings, ART-naïve children are less frequently colonized by *H. pylori* than healthy controls. In particular, low CD4+ counts and advanced disease are associated with a reduced chance of infection [42]. The atypical presentation of opportunistic infections should always be investigated. Multiple biopsies are required as a normal-appearing gross mucosa does not rule out an OI. Chronic nonspecific gastritis with a histology of mononuclear cell infiltrate is a common finding, and CMV is the predominant opportunistic agent in this setting [43].

### 3.2. Children on ART

During the first months on ART, children may frequently complain about nausea, vomiting, dyspepsia, and anorexia, usually associated with old-generation protease inhibitors [44]. In the presence of mild symptoms associated with the initiation of therapy or a change of regime, a watchful wait is recommended, as symptoms tend to resolve within the first weeks of ART. A careful medical history can identify specific problems associated with difficulty with swallowing syrups or large tablets; in such cases, offering an alternative drug formulation when available is a valuable strategy (Table 1).

Hypochloridria, typically associated with untreated HIV infection, reverts with ART and gastroesophageal reflux, *H. pylori* infection, and peptic ulceration are more common in patients on treatment compared with naïve ones. The search for an *H. pylori* infection and an upper endoscopy with multiple biopsies are recommended if symptoms persist or worsen [42,45].

## 4. The Spectrum of Diarrheal Disease in HIV

Diarrheal disease is a hallmark of advanced HIV disease and the presenting symptom in approximately a quarter of new diagnoses. Prior to ART, diarrhea was reported in up to 90% of HIV-positive children living in low-income settings and in 40–80% of those living in high-income countries.

Children living with HIV have a greater risk of moderate-to-severe diarrhea, hospitalization, and death, and are more frequently malnourished compared with uninfected children [46].

The etiology and clinical presentation of diarrheal disease in HIV vary according to the immune status and adherence to antiretroviral treatment. Gastrointestinal infections, HIV-induced enteropathy, small intestinal bacterial overgrowth, malnutrition, and drug-induced diarrhea are common underlying causes.

Thanks to prompt diagnosis and regular access to ART, HIV infection is becoming a chronic disease. In this scenario, other non-infectious etiologies are emerging as causes of chronic diarrhea, including celiac disease, inflammatory bowel diseases, or functional gastrointestinal disorders. In fact, even though the incidence of opportunistic infections has decreased, the total number of patients experiencing diarrhea has not significantly changed, probably as a consequence of the global increase in GI diseases unrelated to immunodeficiency [47]. These should be suspected in any child with persistent symptoms despite ART, good immunological recovery, and the lack of evidence of an underlying infectious cause.

### 4.1. Infectious Diarrhea in ART-Naïve Children and Advanced Disease

In ART-naïve children with diarrhea, investigating infectious causes is essential as even self-limiting infections caused by common GI pathogens tend to be more protracted and severe in HIV-infected individuals with poor immunological function compared with healthy children. A prompt diagnosis and an aggressive treatment are essential.

Causative opportunistic agents in children with HIV span the array of bacteria, viruses, protozoa, and fungi, and are strongly correlated with the level of patients’ immunosuppression. The prevalence of different etiologies varies according to settings, living conditions, and diagnostic techniques used. *Salmonella* spp., *Shigella* spp., enteropathogenic *Escherichia coli* (EPEC), *Cryptosporidium parvum*, *Giardia lamblia*, *Entamoeba histolytica/dispar*, and CMV have all been recently confirmed as predominant agents of chronic diarrhea among treatment-naïve children worldwide [48,49].

#### 4.1.1. Bacterial Etiologies

The incidence of bacterial diarrhea is higher in HIV-positive and treatment-naïve children. *Salmonella* spp. and *Campylobacter jejuni* are common pathogens of the GI tract. Usually, given the mild and self-limiting nature of the infection, no antimicrobial treatment is needed in isolated cases of otherwise healthy children. However, both infections tend to have a more prolonged and severe course in immunocompromised individuals. The risk of non-typhoidal *Salmonella* invasive disease is considerably higher in malnourished children and in advanced HIV disease [50].

*Clostridium difficile* infection is typically associated with the use of antibiotics (mainly third-generation cephalosporins and the combination ampicillin-sulbactam/aminoglycoside), and there is increasing evidence that the risk of infection is greater in HIV patients [51].

MAC usually manifests as a chronic systemic infection with low-grade fever and weight loss, but diarrhea might be present in the case of small bowel involvement.

#### 4.1.2. Viral Etiologies

Viral gastroenteritis, commonly causing an acute and self-limiting disease in immunocompetent children, can represent a significant threat for treatment-naïve or advanced-disease patients. Often, immunocompromised children shed high viral loads in feces, and it is more likely to detect at least one virus in the stools of symptomatic HIV-infected children compared with uninfected controls.

The epidemiology in HIV-positive children reflects the trends seen in healthy children, with group A rotaviruses, enteric adenoviruses, noroviruses, and astroviruses being the leading causes of viral gastroenteritis. Bocavirus, Aichi virus, and sapovirus have recently been described as infectious agents in infected and uninfected HIV-exposed children [52,53,54,55].

However, the rotavirus vaccine is further changing the etiological landscape of viral gastroenteritis, particularly in high- and middle-income countries that have introduced large-scale immunization campaigns. In such settings, norovirus is rapidly becoming the leading agent of medically attended diarrhea; typically, children with HIV present with prolonged and more severe disease and higher norovirus loads that correlate with the degree of immunosuppression [56].

In the advanced stages of HIV, CMV is the leading opportunistic agent of enterocolitis. It can be responsible for severe disease or even intractable diarrhea syndrome in severely immunocompromised children, particularly in infants younger than 12 months of age. Clinical symptoms include chronic, often bloody diarrhea, commonly presenting as a protein-losing enteropathy syndrome with failure to thrive. Bleeding is caused by the ulceration of the GI mucosa, which, in rare cases, may even lead to perforation [57].

#### 4.1.3. Parasitic and Fungal Etiologies

GI parasitosis is reported in more than half of HIV-infected children with diarrhea. The prevalence and the etiology vary according to the geographical setting, socio-economic and nutritional status, and access to safe water and sanitation. Frequently isolated parasites include *Giardia lamblia*, *Entamoeba hystolitica/dispar*, *Strongyloides Stercoralis*, and *Taenia* spp [58,59]. In recent studies, *Giardia* has emerged as a leading cause of parasitic diarrhea. However, the prevalence of giardiasis in HIV-infected children was not higher than in the HIV-exposed but uninfected group. *Giardia* infection can often be asymptomatic. Worsening immunosuppression increases the risk of symptomatic giardiasis. Still, the disease did not seem to have a peculiar or more severe course in children living with HIV than in the otherwise healthy group [48].

Among the opportunistic agents, *Cryptosporidium*, a protozoan parasite classically associated with AIDS, is the most frequent finding in children with diarrhea. The two species more frequently isolated are *C. parvum* (mostly subtype IIa), usually considered a zoonotic disease, and *C. hominis*, almost exclusively transmitted from person-to-person [60,61,62].

*Candida* spp. are frequently isolated from the stools of HIV-positive patients, although in many cases, their presence does not correlate with lower-GI clinical symptoms [63]. *Candida* infection in symptomatic patients is often associated with previous antibiotic use [64].

Immunocompromised children infected by *Microsporidia*, an obligate intracellular fungus, may present with chronic watery diarrhea and failure to thrive or with features of disseminated disease. Several studies have identified *Enterocytozon bieneusi* as the most common species in HIV-positive subjects. The condition, however, is more likely to be seen in adults with advanced disease [65].

#### 4.1.4. Diagnosis of Infectious Diarrhea

When caring for ART-naïve children with acute or chronic diarrhea, a careful review, including family and social history, contact with ill individuals, drugs and travel history, and recent ingestions, is the first step to guide the diagnostic approach.

**Figure 1:**
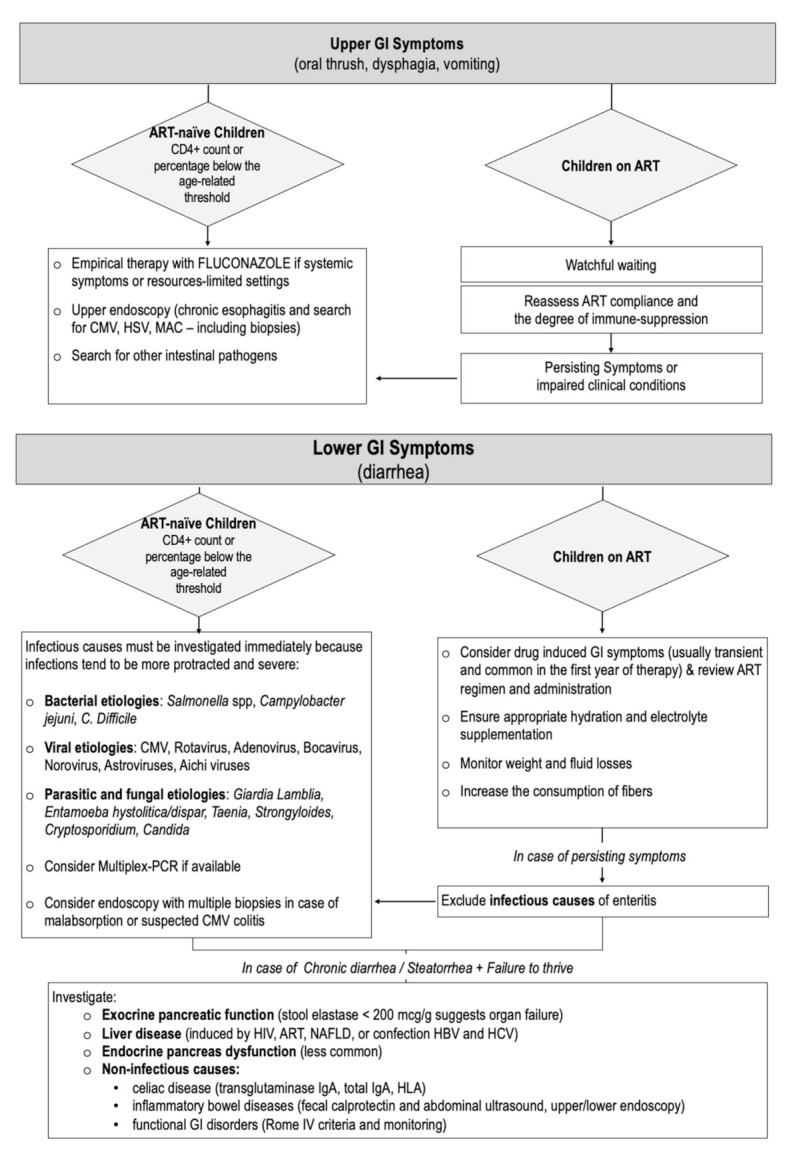
Algorithm for the clinical management of children living with HIV and gastrointestinal disorders.

Obtaining a full blood cell count with differentials, CD4+ counts, and viral load is essential to assess the level of immune suppression and the likelihood of opportunistic infections (Figure 1).

The same approach should be used in children with signs and symptoms suggestive of advanced disease, regardless of the ART status. Treatment adherence should always be investigated.

The diagnostic approach for GI infections should include whole-stool specimens or rectal swabs for the culturing of typical bacteria (i.e., Enterobacteriaceae, *Campylobacter* spp.) and enrichment culturing of atypical bacteria (i.e., *Yersinia*), as well as molecular assays for *Clostridium difficile* A and B toxins, particularly in children with a history of previous hospitalization and antibiotic use.

Direct microscopy for the identification of ova and parasites is recommended on at least three fresh stool samples to increase the chances of detection. The search for *Cryptosporidium* may not be included in standard testing; hence, it is recommended to specifically request it to the laboratory [66,67].

Stool antigen-detection assays are helpful tools, commonly used to detect viral pathogens and parasites such as *Cryptosporidium* and *Giardia*. Both agents can also be identified on endoscopic biopsy or on luminal fluid aspiration during endoscopy [68].

Where available, multiplex PCR panels are highly sensitive and specific tools for the simultaneous detection of several GI pathogens [69]. Most of these tests have not been specifically validated in populations of HIV-positive children; however, they are expected to retain similar diagnostic accuracy compared to other populations of children.

The diagnosis of CMV enterocolitis can be difficult because the virus can remain quiescent for long periods. Serological tests and urine PCR are seldom sufficient for the diagnosis of active disease. The confirmation of infection should be sought through GI endoscopically-guided biopsy, even if the mucosa appears macroscopically normal—suggestive histopathologic findings are intranuclear and intracytoplasmic inclusion bodies. Other techniques, such as tissue immunostaining against CMV antigens, may increase specificity and aid in diagnosis.

Although much less common than other infections and rarely associated with a diarrheal onset, *Mycobacterium tuberculosis* infection should also be considered in the differential diagnosis of children presenting with abdominal pain, malabsorption, intestinal wall thickening, and signs of granulomatous inflammation, especially if the patient is ART-naïve or living in a setting with a high TB prevalence.

Prompt initiation of effective ART and appropriate chemotherapy are the primary initial treatments for most causes of lower GI infections in naïve children and in those with advanced disease or failing on their current regimen. Supportive care with hydration and the correction of electrolyte and blood count abnormalities should always be ensured. In children with chronic diarrhea and poor growth or wasting, after a thorough anthropometric examination and evaluation of intestinal function, nutritional supplementation must always be offered as an integral part of management.

Severe bacterial diarrhea should be treated aggressively. In profoundly immunodeficient children, the prompt use of specific antibiotics should also be considered in the presence of mild symptoms to prevent extraintestinal dissemination of the infection and a prolonged course of the disease [70].

### 4.2. Children on ART and Non-Infectious Causes of Chronic Diarrhea

Temporary drug-induced GI symptoms such as abdominal distension and diarrhea are common, especially in the first year of ART. Up to 29% of patients on any class of antiretrovirals experience mild-to-moderate diarrhea [71].

Diarrhea in children on ART is a frequently overlooked clinical need that contributes to worsening quality of life and poses a risk for sub-optimal adherence to treatment. The evidence base for the management of ART-associated diarrhea in the pediatric and adult population is minimal. The recommended strategy is to use a stepwise approach of nonpharmacologic interventions, including reassuring the patient and the caretakers about the likely mild and transient duration of symptoms, offering supportive measures (hydration and electrolyte supplementation), and, when possible, dietary optimization (increasing the consumption of fibers). Lactose intolerance should be ruled out, especially in older children. Finally, in the presence of invalidating and chronic symptoms that could affect the compliance to ART, modification of the antiretroviral regimen should be considered. To date, Crofelemer, an FDA-approved antidiarrheic agent for adults with HIV/AIDS, is not licensed for use in the pediatric population [72,73] (Table 1).

In the presence of chronic diarrhea in a child on ART with a good immunological status, once infectious causes of enteritis have been ruled out, it is always recommendable to expand the diagnostic workup to non-infectious causes of chronic diarrhea. The etiologies and diagnostic approach do not differ from that of the uninfected child with chronic diarrhea, but it is worth keeping in mind some peculiarities related to HIV infection (Figure 1).

There is no evidence that HIV infection per se increases the risk of developing celiac disease. However, the diagnosis of celiac diseases in HIV-infected children might be difficult due to the possibility of finding false-positive tissue transglutaminase antibodies and HIV-associated villous atrophy on intestinal biopsy. In HIV-infected subjects, HLA-typing may be essential before introducing a gluten-free diet [74,75,76].

In subjects with advanced HIV, some clinical and radiological findings found in severe intestinal infections (such as ulceration or perforation of the mucosa caused by CMV or HSV) or the presence of a thickening of the intestinal wall may require differential diagnosis with inflammatory bowel diseases (IBD). Overall, HIV-infected individuals do not appear to have an increased risk of developing IBD. Indeed, depletion of CD4+ lymphocytes may help in symptom control for patients with inflammatory bowel disease. HIV status was associated with Crohn’s disease remission and with a lower likelihood of relapses [77,78].

Fecal calprotectin is a reliable indicator of gut inflammation, and it is also helpful in children living with HIV. It should be noted, however, that baseline concentrations of fecal calprotectin tend to be higher in younger children with lower CD4+ counts [79].

## 5. Hepatopancreatic Disorders

Morphological changes in the small-intestinal mucosa often appear insufficient to explain the severe malabsorption syndrome observed in many children [80]. In fact, one third of children with advanced disease show laboratory evidence of pancreatic insufficiency, and in these patients, there is a clear correlation between pancreatic dysfunction and fat malabsorption. Hence, impaired exocrine pancreatic function should be ruled out in any patient with chronic diarrhea/steatorrhea and failure to thrive. The measurement of stool elastase is a useful screening tool, more sensitive than fecal fat. Concentrations of elastase <200 µg/g dry feces suggest exocrine pancreatic insufficiency, although levels have to be interpreted carefully if the stools are watery [81]. Nutrient malabsorption increases the progression of HIV disease and is a strong negative prognostic factor. Any child with pancreatic dysfunction should be considered for pancreatic enzyme supplementation [82]. Endocrine pancreas dysfunction is less common, but secondary insulin resistance is present in up to 10% of children on ART with dyslipidemia and a high body mass index [83,84].

Liver disease is the most common cause of death in HIV-infected adults in most high-income settings, accounting for up to 18% of all deaths; on the contrary, in the pediatric population, its incidence and influence on mortality seem to be marginal. Liver injury may result from HIV infection itself but also from ART toxicity or comorbidities, including coinfection with hepatitis B and C viruses (HBV and HCV) and nonalcoholic fatty liver disease (NAFLD). Isolated hepatomegaly with normal or slightly raised transaminases (ALT less than three times the normal upper limit) is a relatively common finding in children living with HIV, which could be associated with nutritional deficiencies or NAFLD [85].

The global prevalence of HIV-HBV coinfection has been re-shaped by immunization campaigns and newborn prophylaxis; hence, it shows great variability (1–49%) according to the examined setting. It is essential to screen all children for HBV before the start of ART and, in the case of coinfection, it is recommendable to choose a lamivudine-sparing regimen as the use of this drug could select HBV-resistant strains [86]. HBV/HIV coinfection exposes children to a higher risk of hepatic disease and fibrosis; however, although in the adult population there is a well-established association between HIV/HBV coinfection and the risk of hepatocellular carcinoma (HCC), limited data are available for the pediatric population. It is estimated, however, that up to 40% of children with coinfection develop HCC later in life. Recent data from a large north American cohort of adults living with HIV have shown an increased risk of developing HCC even in the absence of HBV, but with the exception of sporadic case reports, little is known about the association between perinatally-acquired HIV and HCC [87,88,89,90].

Furthermore, HIV/HCV-coinfected children are at greater risk of advanced fibrosis in late adolescence. To date, direct acting antivirals (DAAs) have been approved for the treatment of all HCV-infected children aged 12 to 17 years. DAAs are a safe and effective option in perinatally infected HIV/HCV patients; therapy should be started early to prevent disease progression [91,92].

### Children on ART

In children on ART, pancreatitis and hepatitis are infrequent but potentially severe complications associated with treatment. The risk of ART-associated pancreatitis is increased in children with advanced disease, in children concomitantly taking other drugs such as cotrimoxazole, and in those with a history of hypertriglyceridemia. In the case of persistent abdominal pain in any child on ART, especially if associated with other symptoms such as vomiting, it is always recommended to measure serum lipase and pancreatic amylase. The diagnosis is confirmed in the presence of significantly raised pancreatic enzymes and suggestive clinical symptoms; the management of the acute episode does not differ from that of the non-HIV infected child, bearing in mind that the offending antiretrovirals should be immediately stopped and replaced with other drugs [93,94] (Table 1).

ART-induced liver damage is reported in less than 10% of children and was more frequently seen in the past when nevirapine was the mainstay of treatment (Table 1). Liver damage may present with a variety of clinical forms, from idiosyncratic hepatotoxicity to hypersensitivity reactions, where hepatitis (often severe) is characteristically associated with skin rash and lactic acidosis. Integrase inhibitors are rarely associated with acute liver or pancreatic damage, although the association between dolutegravir and the onset of hyperglycemia is currently being investigated in adult populations [95,96,97]. The isolated elevation of liver enzymes may be observed in children on ART and this requires monitoring, as well as a liver ultrasound assessment to rule out steatosis, commonly associated with zidovudine. Non-invasive tools for liver function assessments, such as the AST-to-platelet ratio index (APRI) and fibrosis-4 (FIB-4), are being increasingly used in the pediatric population as they reduce the need for biopsy. In most cases, however, long-term treatment is not associated with significant alterations of liver function [98,99].

## 6. Conclusions

Advances in therapy have transformed the spectrum of HIV in pediatric patients, including the incidence, severity, and outcomes of HIV-associated GI complications. Intestinal involvement is present in virtually all children with HIV infection. In high-prevalence settings, more than half of patients with advanced disease present with GI symptoms, from mild infectious diarrhea to severe GI impairment, malabsorption, and failure to thrive. Gastrointestinal disorders do not spare children on ART, particularly in the initial months of therapy. Lower abdominal symptoms have been reported in more than a third of patients, and may limit ART adherence, although this they are usually mild and transient. A correct framing of the problem is necessary in order to minimize therapy switches, while optimizing the quality of life of children on ART. ART-associated pancreatitis and hepatitis are rare but potentially severe adverse events.

The literature about GI diseases in children living with HIV is sparse and the purpose of this review article was to provide a quick updated reference for management strategies to improve evidence-informed clinical practices, particularly for the child on ART. The lack of a systematic reviewing approach and the subjective weighing of the studies included might be considered the main limitation of this review article. However, the consideration given to the pathophysiology of intestinal damage in children who are either ART-naïve or who are not, along with the global epidemiology of opportunistic infections, make it an up-to-date resource that any HIV clinician dealing with pediatric patients in any setting can benefit from.

Future perspectives should prioritize the evaluation of the diagnostic yield of modern techniques to investigate the epidemiology of viral infections in children living with HIV on ART, the collection of new evidence about GI features in children living in resource-limited settings, and the optimization of the management of GI side-effects, as well as their monitoring in children receiving new ART combinations (dual therapy, fixed-doses combinations).

## Figures and Tables

**Table 1:** Gastrointestinal adverse events associated with antiretroviral drugs.

Symptoms Upper GI	Antiretrovirals	Characteristics	Management
***Nausea, Emesis***	All ARV, particularly PI	Incidence < 15% Usually mild, during the first months on ART, then resolves spontaneously.	- Reassure caretakers. - Recommend taking PI with food. - Change drug formulation when available (smaller tablet size, syrups). - If persistent/severe vomiting, consider antiemetics and switching to a different regimen.
**Lower GI**			
***Diarrhea, Abdominal Discomfort***	All ARV, particularly PI	Frequent, seen in up to one third of patients during the first months on ART. Mild diarrhea, non-bloody, and usually non-watery. Lactose intolerance may be triggered by drugs (Some FDC tablets contain lactose)	- Reassure caretakers. - Treat dehydration when present. In older children, dietary modifications may help. - If persistent/chronic, rule out infections and other organic causes. - In case of persistent/severe symptoms or weight loss, consider switching to a different regimen.
**Liver**			
***Hepatitis***	All ARV, particularly NNRTI (NVP and EFV)	Incidence 1–9% (severe hepatitis and hypersensitivity reactions <1%) Liver involvement can range from asymptomatic increase in liver enzymes to severe hepatitis with lactic acidemia and rash in hypersensitivity reactions.	- Rule out other infections (HAV, HBV, HCV, EBV, CMV). - Asymptomatic hypertransaminasemia: monitor and discontinue treatment if ALT > 5×UNL. - Symptomatic hepatitis: suspend ART and other possibly hepatotoxic drugs. If on NVP, discontinue permanently.
***Isolated Hyperbilirubinemia (indirect)***	ATV	Early-onset, usually in the first months of therapy. ATV-associated indirect hyperbilirubinemia is usually an isolated finding with normal transaminases.	- Reassure caretakers. - ATV discontinuation not required.
***Steatosis***	ZDV	Rare in pediatric patients, usually develops after years on treatment.	
**Pancreas**			
***Pancreatitis***	NRTI, PI	<2% Unpredictable onset, usually after months of ART. Acute pancreatitis with significantly raised serum lipase and amylase.	- Stop the offending drug and substitute with other ARV - Symptomatic management.

Adapted from [100]. Abbreviations: ARV, antiretrovirals; PI, protease inhibitors; NNRTI, non-nucleoside reverse transcriptase inhibitors; NVP, nevirapine; EFV, efavirenz; ATV, atazanavir; ZDV, zidovudine; NRTI, nucleoside reverse transcriptase inhibitors; FDC, fixed dose combination; UNL, normal upper limit.

## Data Availability

Not applicable.
